# Coordinate regulation of systemic and kidney tryptophan metabolism by the drug transporters OAT1 and OAT3

**DOI:** 10.1016/j.jbc.2021.100575

**Published:** 2021-03-21

**Authors:** Jeffry C. Granados, Anne Richelle, Jahir M. Gutierrez, Patrick Zhang, Xinlian Zhang, Vibha Bhatnagar, Nathan E. Lewis, Sanjay K. Nigam

**Affiliations:** 1 Department of Bioengineering, University of California San Diego, La Jolla, California, USA; 2 Department of Pediatrics, University of California San Diego, La Jolla, California, USA; 3 Department of Biology, University of California San Diego, La Jolla, California, USA; 4 Division of Biostatistics and Bioinformatics, Department of Family Medicine and Public Health, University of California San Diego, La Jolla, California, USA; 5 Department of Family and Preventative Medicine, University of California San Diego, La Jolla, California, USA; 6 Novo Nordisk Foundation Center for Biosustainability at UC San Diego, University of California San Diego, La Jolla, California, USA; 7 Department of Medicine, University of California San Diego, La Jolla, California, USA

**Keywords:** drug transport, kidney, kidney metabolism, tryptophan, gut microbiome, uremic toxins, chronic kidney disease, organ crosstalk, drug transporters, xenobiotic, AhR, aryl hydrocarbon receptor, CKD, chronic kidney disease, DMI, drug–metabolite interaction, ESI, electrospray ionization, HEK, human embryonic kidney, ILA, indolelactic acid, KO, knockout, RP/UPLC/MS, reverse-phase ultraperformance liquid chromatography–mass spectrometry

## Abstract

How organs sense circulating metabolites is a key question. Here, we show that the multispecific organic anion transporters of drugs, OAT1 (SLC22A6 or NKT) and OAT3 (SLC22A8), play a role in organ sensing. Metabolomics analyses of the serum of *Oat1* and *Oat3* knockout mice revealed changes in tryptophan derivatives involved in metabolism and signaling. Several of these metabolites are derived from the gut microbiome and are implicated as uremic toxins in chronic kidney disease. Direct interaction with the transporters was supported with cell-based transport assays. To assess the impact of the loss of OAT1 or OAT3 function on the kidney, an organ where these uptake transporters are highly expressed, knockout transcriptomic data were mapped onto a “metabolic task”-based computational model that evaluates over 150 cellular functions. Despite the changes of tryptophan metabolites in both knockouts, only in the *Oat1* knockout were multiple tryptophan-related cellular functions increased. Thus, deprived of the ability to take up kynurenine, kynurenate, anthranilate, and N-formylanthranilate through OAT1, the kidney responds by activating its own tryptophan-related biosynthetic pathways. The results support the Remote Sensing and Signaling Theory, which describes how “drug” transporters help optimize levels of metabolites and signaling molecules by facilitating organ cross talk. Since OAT1 and OAT3 are inhibited by many drugs, the data implies potential for drug–metabolite interactions. Indeed, treatment of humans with probenecid, an OAT-inhibitor used to treat gout, elevated circulating tryptophan metabolites. Furthermore, given that regulatory agencies have recommended drugs be tested for OAT1 and OAT3 binding or transport, it follows that these metabolites can be used as endogenous biomarkers to determine if drug candidates interact with OAT1 and/or OAT3.

The organic anion transporters OAT1 (SLC22A6, originally NKT (1)) and OAT3 (SLC22A8, originally ROCT (2)) are found in the proximal tubule of the kidney and choroid plexus of the brain and control the physiological distribution and elimination of numerous drugs and environmental toxins as well as endogenous metabolites such as α-ketoglutarate, prostaglandins, and urate (3, 4). In addition, OATs help regulate levels of ingested natural products and gut microbiome-derived compounds (*e.g.*, epicatechin, 3-indoxyl sulfate, p-cresol sulfate) (5, 6).

OAT1 and OAT3, members of the SLC22 family, are among the best-known multispecific drug transporters. This group of drug transporters includes other members of the SLC (solute carrier) and the ABC (ATP-binding cassette) superfamilies (7, 8). There is extensive literature on the pharmaceutical and toxicological roles of these transporters, yet the endogenous functions of these evolutionarily conserved proteins, as well as the regulatory network in which they participate, are only beginning to be characterized (9). These multispecific transporters influence many aspects of physiology and pathophysiology, likely by functioning in combination with mono- or oligo-specific transporters. Three well-characterized pathophysiological examples are the roles of OAT1, OAT3, URAT1, and ABCG2 in gout (10), the role of OAT1 and OAT3 in the accumulation of uremic toxins associated with chronic kidney disease (CKD) (11, 12), and the SLC22 family in acute kidney injury (13).

Because of the vast array of small organic anion compounds transported by OAT1 and OAT3, a wide range of xenobiotics and endogenous molecules can conceivably compete for access to and renal clearance by these OAT transporters (14, 15). This has important implications because many of the metabolites, including those arising from the gut microbiome and transported by OAT1 and OAT3, regulate endogenous physiology and have been linked to the development of clinical disorders, such as CKD, metabolic syndrome, and diabetes (14, 16, 17). For example, the amount of CMPF (3-carboxy-4-methyl-5-propyl-2-furanpropanoic acid), a substrate of OAT3, in the circulation has been related to diabetes. CMPF is a furan fatty acid metabolite that is found in fish oils, vegetable oils, butters, and other foods and perturbs pancreatic β cell function, leading to glucose intolerance (18, 19). The potential consequences of transporter-level competition between a drug and the transported endogenous metabolite include: (a) altered intracellular concentrations of metabolites because a transported drug blocks metabolite entry into the cell; (b) altered serum concentrations of the drug, the metabolite, or both, leading to increased half-lives and/or metabolite levels; and (c) altered systemic metabolism arising from distal cascading effects on multiple metabolic pathways that depend upon OAT1 or OAT3 or both.

We applied a systems biology approach that employed metabolomic and transcriptomic data from *Oat1* and *Oat3* knockout (KO) mice to analyze the major metabolic functions that are influenced by OAT1 and OAT3. The serum metabolomic data from the *Oat1* and *Oat3* KO mice represented the impact these transporters have on endogenous systemic metabolism. We used the transcriptomic data from the kidney, which is the site of expression of the encoding transporter genes, to evaluate the activity of hundreds of metabolic functions using metabolic task analysis. In doing so, we were able to complement an understanding of extracellular metabolite changes due to the loss of the OAT1 or OAT3 transporters with an analysis of affected intracellular metabolic pathways.

Together, the multi-omics approach and systems biology analysis used here provide a portrait of the local and systemic metabolic and signaling pathways modulated separately and jointly by OAT1 and OAT3. We show that OAT1 not only regulates tryptophan metabolism systemically, but it also plays a key role in the tryptophan metabolic pathways inside the tissue where it is found. Thus, our results indicate that studies of new drug entities should not only consider drug–metabolite interaction (DMI) effects in the serum but also evaluate potential shifts in cellular metabolism due to loss of metabolite influx through competition at the level of the transporter. In support of the human relevance of our work, we show that many of the alterations in serum levels of tryptophan metabolites seen in the knockout mice are also observed in humans after administration of probenecid, a drug used to treat gout that inhibits OAT1 and OAT3.

## Results

### Summary of overall approach

**Figure 1:**
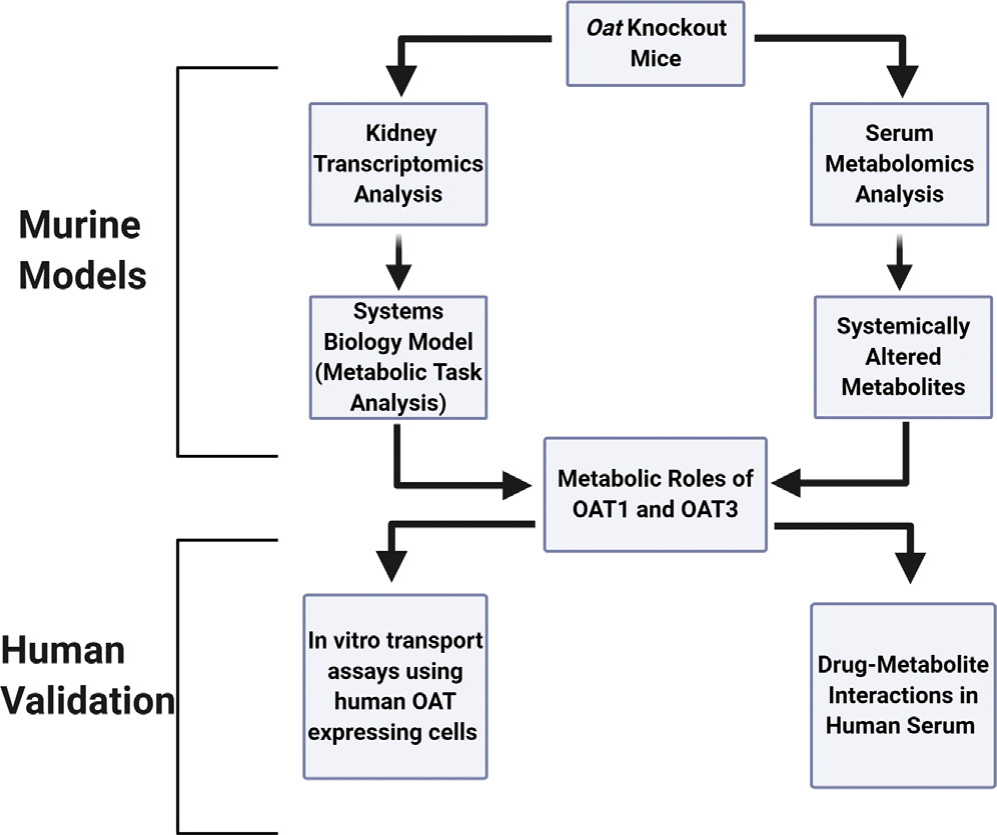
**Strategy for determining metabolic role of drug transporters.** By combining tissue-specific transcriptomic data with serum metabolomic data, the local and systemic roles of the OAT1 and OAT3 transporters can be determined. Using this framework, we investigated the role of each transporter in cellular metabolism of the relevant tissue (kidney), as well as the role the transporters play in controlling metabolite concentrations in the serum. These studies were supported with clinically relevant human data as described in Results.

The SLC22 gene family (OATs, OCTs, OCTNs) encodes for transporters that participate in the uptake of many unique compounds across several tissues, though much of the research has focused on a handful of members (SLC22A1, SLC22A2, SLC22A6, SLC22A8) that handle many common drugs (20, 21). However, these genes are highly conserved, with orthologs in fly, worm, fish, and sea urchin (22); this implies they have important physiological roles beyond handling drugs. To determine the endogenous roles of OAT1 and OAT3 in controlling tissue-level and organismal-level metabolism independent from their well-known roles as drug and toxin transporters, we analyzed tissue-specific transcriptomic data and serum metabolomic data (Fig. 1). We compared data from mice genetically deficient in either *Oat1* or *Oat3* and their wild-type controls. These data sets have previously been examined from a chemoinformatic perspective (23) and to provide a broad overview of affected pathways (24), but here we place an emphasis on their specific role in the disposition of tryptophan metabolites. Because these two transporters are found in the kidney, we analyzed kidney transcriptomic data using Metabolic Task Analysis, a systems biology method that groups genes according to their coordinated roles in the biosynthesis of a limited set of key metabolite intermediates from diverse metabolite inputs (see Methods section for more details). We supported these studies with *in vitro* transport assays; we also support the clinical relevance of our findings with human metabolomics data.

### Known characteristics of *Oat1* KO and *Oat3* KO mice

Almost all the characteristics of mice deficient in *Oat1* or *Oat3* are similar to those of wild-type mice (25, 26, 27) (Table 1). The knockout mice are viable with survival comparable to that of wild-type mice. Moreover, they display no apparent developmental or growth abnormalities, and histological and physiological examination of these various knockouts (aged ~2–8 months) revealed little or no differences from wild type, other than the *Oat3* KO mice having a slightly lower blood pressure (28). A trend toward hepatic lipid deposition in the *Oat1* KO has also been noted but only for very old mice (29).

**Table 1:** Summary of known physiological characteristics of *Oat1* KO and *Oat3* KO mice

Characteristic	*Oat1 KO*	*Oat3 KO*
Viable	✓	✓
Fertile	✓	✓
Histological abnormalities	None observed	None observed
Anatomic abnormalities	None observed	None observed
Body weight	Comparable to wildtype	Comparable to wildtype
Renal function	Comparable to wildtype	Comparable to wildtype
General metabolic parameters	Comparable to wildtype	Comparable to wildtype
General physiological parameters	Comparable to wildtype	Lower blood pressure
Lifespan	Comparable to wildtype	Comparable to wildtype

A check mark indicates no differences from wild-type animals. General metabolic parameters include oxygen consumption, carbon dioxide production, food and water intake, and locomotor activity. General physiological parameters include body weight and plasma chemistries. Data are from (25, 26, 27, 28).

These transporter knockout mice have also been characterized from a pharmacological and toxicological standpoint (Table 2). OAT1 and OAT3 play important roles in the handling of various drugs and toxins, both environmental and endogenously produced (30, 31, 32, 33, 34, 35, 36, 37, 38). Both knockout mice have altered responses to loop and thiazide diuretics due to the inability of these drugs to access the lumen of the nephron—a process dependent upon OAT1-or OAT3-mediated uptake into the proximal tubule cell. *Oat1* KO animals are protected against mercury-induced nephrotoxicity due to a lack of kidney uptake of mercury conjugates. Both mice accumulated uremic toxins in the serum and exhibited decreased secretion of uric acid. *Ex vivo* analysis of tissues from either *Oat1* KO or *Oat3* KO mice shows reduced uptake of several antivirals.

**Table 2:** Drugs and toxins with altered elimination in *Oat1* KO and *Oat3* KO mice or reduced uptake in *ex vivo* or *in vivo* experiments with tissues from these mice

Drug or toxin	*Oat1 KO*	*Oat3* KO	*In vivo* or *ex vivo*	Reference
Antivirals	✓	✓	*Ex vivo*	(36)
Antibiotics	✓	✓	*In vivo*	(30)
Diuretics	✓	✓	*In vivo*	(32)
Fluoroquinolone antibiotics		✓	*In vivo*	(31)
Mercury	✓		*In vivo*	(33)
Uremic Toxins	✓	✓	*In vivo*	(34, 35)
Uric acid	✓	✓	*In vivo*	(25)

A check indicates altered handling or elimination based on cited reference.

We have reported that *Oat1*-deficient animals may have reduced expression of OAT3 (39). However, a number of studies have indicated that the functional impact of this is modest at best in the basal state. For example, the *Oat1* knockout kidneys have markedly reduced PAH transport, but not apparent loss of transport of the OAT3 substrate, estrone sulfate (26). Chemoinformatic analysis has identified sets of molecular properties that distinguish metabolites altered in *Oat1* compared with the *Oat3* deficient mice (23). Furthermore, embryonic kidney organ cultures from both lines of mice transported different sets of antivirals (36, 37, 38). There also are differences in the types of uremic toxins found in the *Oat1 versus* the *Oat3* KO (34).

### Tryptophan metabolism is altered systemically in *Oat1* and *Oat3* KO mice

Published metabolomic analyses of *Oat1* KO and *Oat3* KO mice suggest physiologically important alterations in the handling of endogenous metabolites and gut microbiome products, including some uremic toxins (34, 35). Here, we analyzed 731 metabolites of known identity in the serum collected from control and *Oat1* KO mice. Likewise, 611 metabolites of known identity were detected in the serum from control and *Oat3* KO mice.

For *Oat1* KO mice, we found that 63 metabolites in the Amino Acid Superpathway (Supplementary Table S1) were significantly increased (*p* ≤ 0.05, 54 metabolites, among which 12 metabolites had fold changes higher than 3) or trending toward significantly increased (0.05 ≤ *p* ≤ 0.10, nine metabolites), suggesting that they are either OAT1 substrates or that their serum concentrations depend on OAT1. For *Oat3* KO mice, we found that ten metabolites in the Amino Acid Superpathway were significantly increased, while 11 were trending toward significantly increased (Supplementary Table S2). As a complement to the *p*-values, we also calculated Cohen's d, which showed large effect sizes (ranging from 1.76 to 4.17) for tryptophan metabolites. Consistent with other pharmacological and physiological data obtained from the OAT1 and OAT3 knockout mice or their tissue indicating important functional differences (23, 36, 38), comparison of changes in serum metabolites in the Amino Acid Superpathway between *Oat1* KO (63 metabolites) and *Oat3* KO (21 metabolites) mice revealed only ten overlapping metabolites in these subsets (Supplementary Table S3).

**Figure 2:**
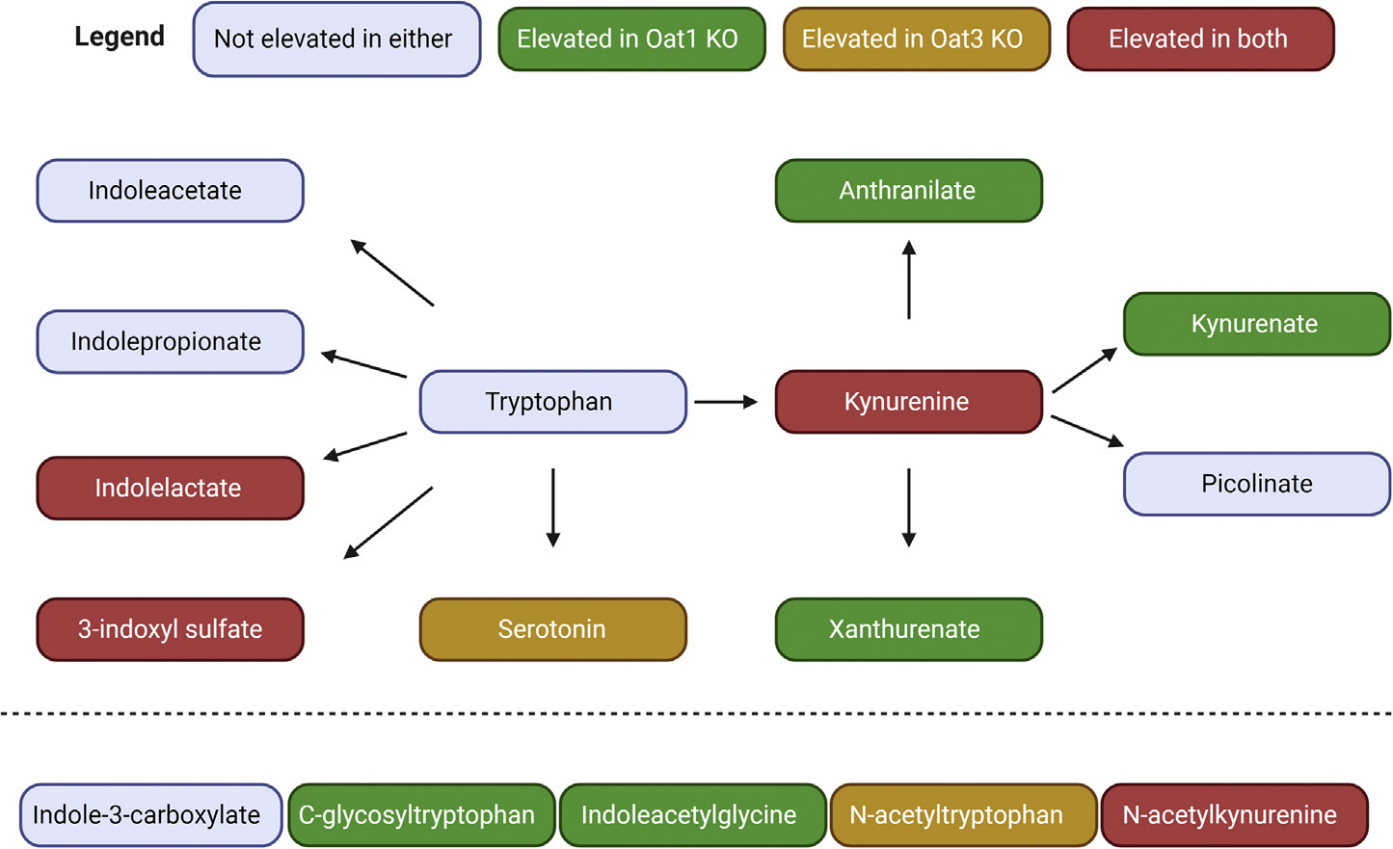
**Systemic tryptophan metabolites regulated by OAT1, OAT3, or both transporters.** The metabolites measured in both knockout animals are shown in the figure with an edge between two nodes representing one or several reactions that lead to production of the metabolite. Note that, apart from the four common metabolites depicted, different tryptophan metabolites accumulate in the *Oat1* KO and the *Oat3* KO. The five metabolites at the bottom have yet to be placed in the biochemical map but are known to be tryptophan derivatives. Statistical significance was determined by Welch's *t*-test. Individual fold changes for the altered metabolites are as follows: Indolelactate (OAT1: 2.63, OAT3: 2.37), 3-indoxyl sulfate (OAT1: 4.29, OAT3: 2.88), Serotonin (OAT3: 1.75), Kynurenine (OAT1: 1.58, OAT3: 1.51), Kynurenate (OAT1: 2.51), Anthranilate (OAT1: 2.67), Xanthurenate (OAT1: 5.29), C-glycosyltryptophan (OAT1: 1.51), Indoleacetylglycine (OAT1: 2.59), N-acetyltryptophan (OAT3: 2.68), N-acetylkynurenine (OAT1: 1.68, OAT3: 2.11).

**Figure 3:**
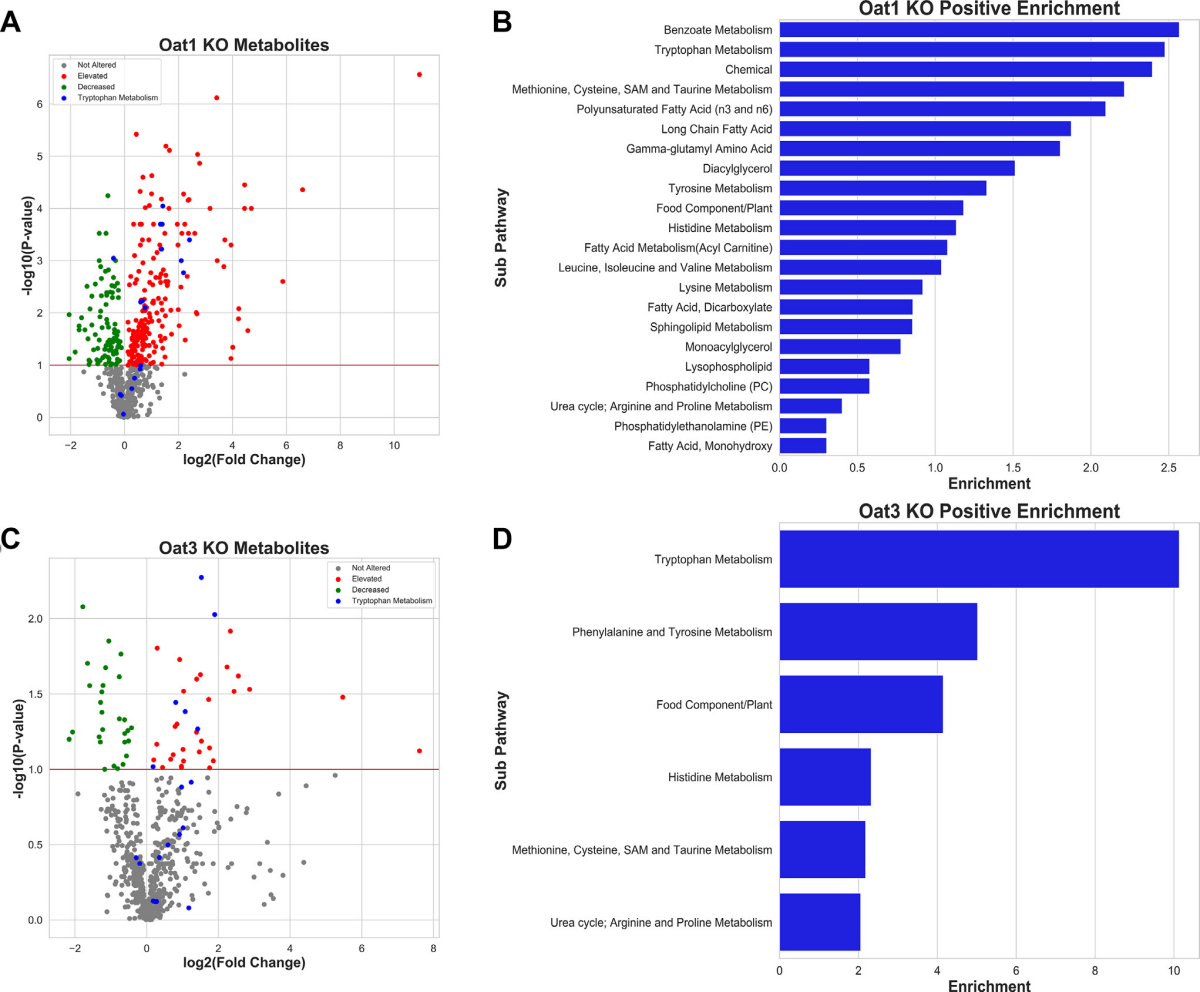
**Tryptophan metabolism is one of the most altered pathways in both knockout mice.***A*, volcano plot for the *Oat1* KO (n = 5) showing the tryptophan metabolites against all other measured metabolites. *B*, tryptophan metabolism is the second most enriched pathway for significantly elevated metabolites. *C*, volcano plot for the *Oat3* KO (n = 3) showing the tryptophan metabolites against all other measured metabolites. *D*, tryptophan metabolism is the most enriched pathway for significantly elevated metabolites based on metabolon subpathway analysis.

Each metabolite was classified using Metabolon's software into one of eight Superpathways (Amino Acid, Carbohydrate, Cofactors and Vitamins, Energy, Lipid, Nucleotide, Peptide, Xenobiotics) and one of 90 Subpathways. Of the 17 metabolites that significantly accumulated in the serum of both the *Oat1* KO and *Oat3* KO animals, four belonged to the Tryptophan Metabolism Subpathway, indicating that these two transporters have an important shared role in regulation of systemic tryptophan metabolism (Fig. 2). Independently, Tryptophan Metabolism was among the most enriched Subpathways for both knockout models (Fig. 3). Sixteen tryptophan metabolites were measured in the serum of both mice, and 12 were significantly altered in at least one group. The *Oat1* KO mice had 11 metabolites elevated, including one (N-formylanthranilate) that was not measured in the serum of the *Oat3* KO (Table 3). Analysis of the *Oat3* KO revealed that six of the 16 metabolites were significantly or trending toward significantly altered. Some of these metabolites, including those arising from bacteria in the gut microflora, are known to have distal effects on several other organs. For example, 3-indoxyl sulfate is associated with the progression of kidney disease and the uremic syndrome (12, 40). The data indicate that OAT1 and OAT3 work together to regulate systemic tryptophan metabolism, and yet they also have specific functions within tryptophan metabolism by handling distinct sets of metabolites affecting different biochemical reactions.

**Table 3:** Altered serum abundance of components of the tryptophan metabolism subpathway in *Oat1* KO or *Oat3* KO mice

Metabolites	*Oat1* KO fold change	Altered in *Oat1* KO serum (*p*-value)	Existing OAT1 *in vitro* support (reference)	*Oat3* KO fold change	Altered in *Oat3* KO serum (*p*-value)	Existing OAT3 *in vitro* support (reference)	Signaling role (reference)
3-Indoxyl sulfate	4.29	✓ (0.001)	(35)	2.88	✓ (0.005)	(35, 69)	AhR activator (62)
5-Hydroxyindoleacetate	NM	NM		1.28			
Anthranilate	2.67	✓ (0.000)	(70)	1.21		(70)	
C-glycosyltryptophan	1.51	✓ (0.006)		0.82			
Indole-3-carboxylate	1.20			1.88			
Indoleacetate	1.29		(69)	2.37		(69)	AhR activator (71)
Indoleacetylglycine	2.59	✓ (0.001)		1.96			
Indolelactate	2.63	✓ (0.000)	This paper	2.37	✓ (0.009)	This paper	
Indolepropionate	0.89			2.25			PXR, NF-kB signaling inhibitor (72, 73)
Indolepropionylglycine	1.49			NM	NM		
Kynurenate	2.51	✓ (0.000)	(70)	1.51		(70)	AhR, GPCR, NMDAR activator (74, 75, 76)
Kynurenine	1.58	✓ (0.006)	(35)	1.13	✓ (0.096)		AhR activator (77)
N-acetylkynurenine	1.68	✓ (0.008)		2.11	✓ (0.041)		Potential AhR activator (78)
N-acetyltryptophan	0.97			2.68	✓ (0.054)		GPCR activator (79)
N-formylanthranilate	4.54	✓ (0.002)		NM	NM		
Picolinate	1.51		(70)	2.02		(70)	
Serotonin	0.92			1.75	✓ (0.036)		AhR, GPCR activator (80, 81)
Tryptophan	0.79	✓ (0.001)		0.87			
Xanthurenate	5.29	✓ (0.000)	(35, 70, 82)	1.17		(70, 82)	AhR activator (74)

Of those that are altered, only tryptophan decreased in abundance. ✓ represents statistical significance. The *p*-values by Welch's *t*-test are in parentheses. NM is not measured. All altered metabolites below had effect sizes (Cohen's d) between 1.74 and 4.17.

### Evaluation of drug–metabolite interactions in humans involving an OAT-inhibiting drug and tryptophan metabolites

**Figure 4:**
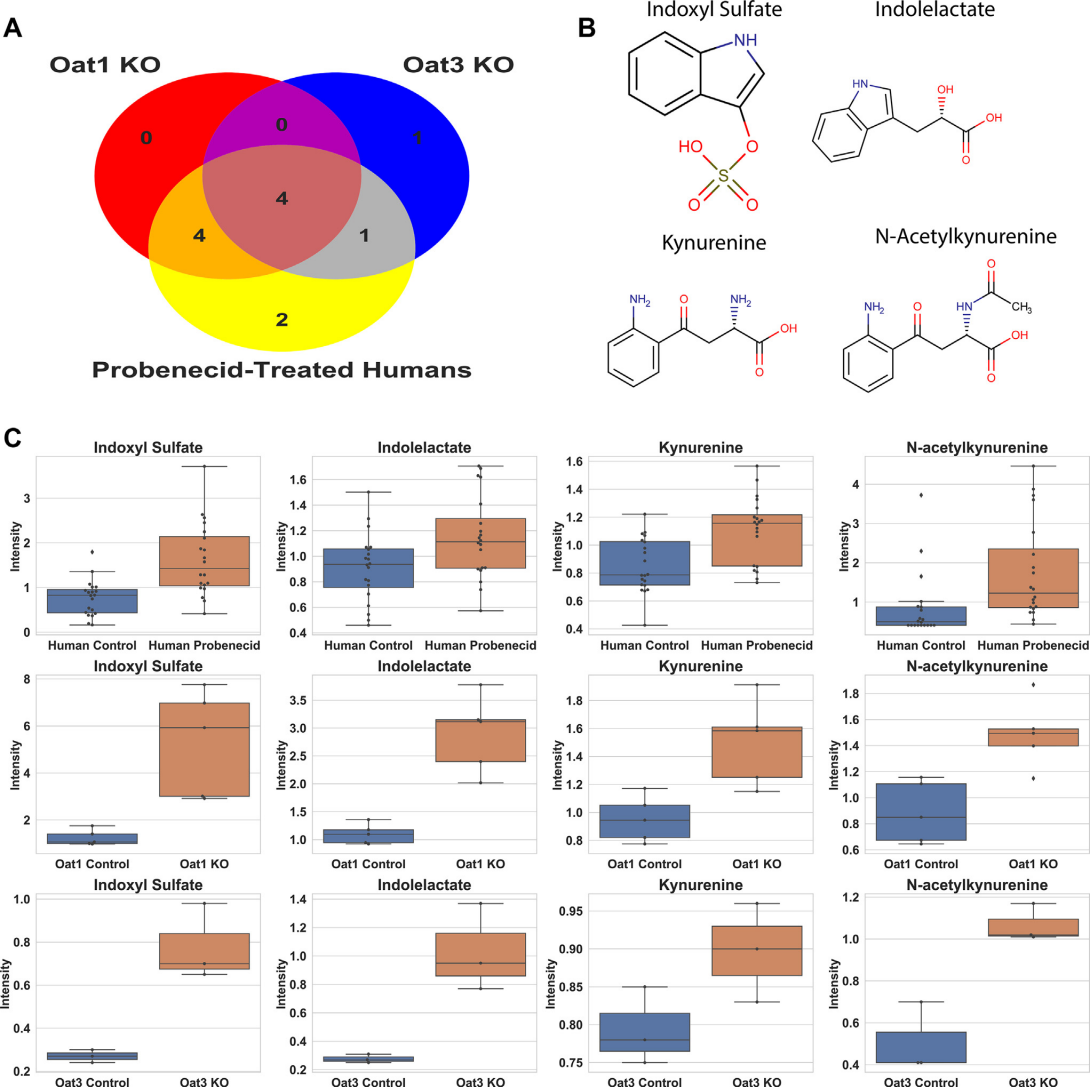
**Tryptophan metabolites were elevated in *Oat* knockout mice and probenecid-treated humans**. *A*, Venn diagram of significantly altered tryptophan metabolites in knockout mice and humans treated with probenecid. Of the 14 metabolites common to all three platforms, 12 were significantly elevated in at least one of the experiments. Four metabolites were significantly elevated in each experiment. *B*, chemical structures of metabolites elevated in each experiment: 3-indoxyl sulfate, indolelactate, kynurenine, and N-acetylkynurenine. *C*, boxplots for each metabolite in the probenecid-treated humans (n = 20), *Oat1* KO mice (n = 5), and *Oat3* KO mice (n = 3). Lines in boxplots indicate the median.

Probenecid, a drug used to treat gout, was administered to humans to determine the short-term impact of a high-affinity OAT-binding drug on the levels of circulating metabolites. Probenecid has three well-established targets: SLC22A12 (URAT1, Rst), OAT1, and OAT3, all of which are closely related genes in the organic anion transporter group of the SLC22 family of solute carriers. URAT1 is a uric acid transporter located on the apical membrane (urine-facing side) of the proximal tubule, so OAT1 and OAT3, which are multispecific and expressed on the blood-facing side of the proximal tubule, are expected to exert a more profound effect on the serum metabolome. Comparisons of metabolic measurements on the blood before and 5 h after dosage show major changes to the Tryptophan Metabolism Subpathway (Table 4). Due to differing platforms, there were 22 metabolites in this Subpathway, 16 of which were significantly altered. Fourteen metabolites were measured across all three platforms and revealed several commonalities between the knockout mice and probenecid-treated humans (Fig. 4). For example, increases in 3-indoxyl sulfate, kynurenine, N-acetylkynurenine, and indolelactate were observed in both knockout mice groups and humans. However, there was more overlap between the *Oat1* KO mouse and humans treated with probenecid. All eight metabolites that were significantly elevated in the *Oat1* KO were also significantly elevated in the drug-treated humans. Only serotonin, which was elevated in the *Oat3* KO mouse, was not altered in humans.

**Table 4:** Altered serum abundance of components of the tryptophan metabolism subpathway in probenecid-treated humans

Metabolite	Fold change	*p* value	Significantly altered in humans	Significantly altered in *Oat1* KO	Significantly altered in *Oat3* KO
3-Indoxyl sulfate	2.44	0.0000	✓	✓	✓
5-Hydroxyindole sulfate	3.8	0.0000	✓	NM	NM
5-Hydroxyindoleacetate	2.28	0.0199	✓	NM	
6-Bromotryptophan	0.92	0.1104		NM	NM
8-Methoxykynurenate	2.76	0.0000	✓	NM	NM
C-glycosyltryptophan	0.89	0.0138	✓	✓	
Indole-3-carboxylate	1.08	0.9409			
Indoleacetate	1.45	0.0019	✓		
Indoleacetoylcarnitine	0.94	0.2267		NM	NM
Indoleacetylglutamine	4.35	0.0000	✓	NM	NM
Indolelactate	1.29	0.0004	✓	✓	✓
Indolepropionate	1.27	0.1012			
Kynurenate	2.53	0.0000	✓	✓	
Kynurenine	1.32	0.0001	✓	✓	✓
N-acetylkynurenine (2)	2.57	0.0000	✓	✓	✓
N-acetyltryptophan	1.72	0.0002	✓		✓
N-formylanthranilate	2.05	0.0000	✓	✓	NM
Picolinate	1.18	0.0999	✓		
Serotonin	1.19	0.6392			✓
Tryptophan	0.82	0.0004	✓	✓	
Tryptophan betaine	19.09	0.9247		NM	NM
Xanthurenate	2.72	0.0000	✓	✓	

Many of the changes observed in the knockout mice are reflected in the humans treated with probenecid. ✓ represents statistical significance. NM is not measured.

### Mice treated with an OAT-inhibiting drug show alterations in tryptophan metabolism

**Figure 5:**
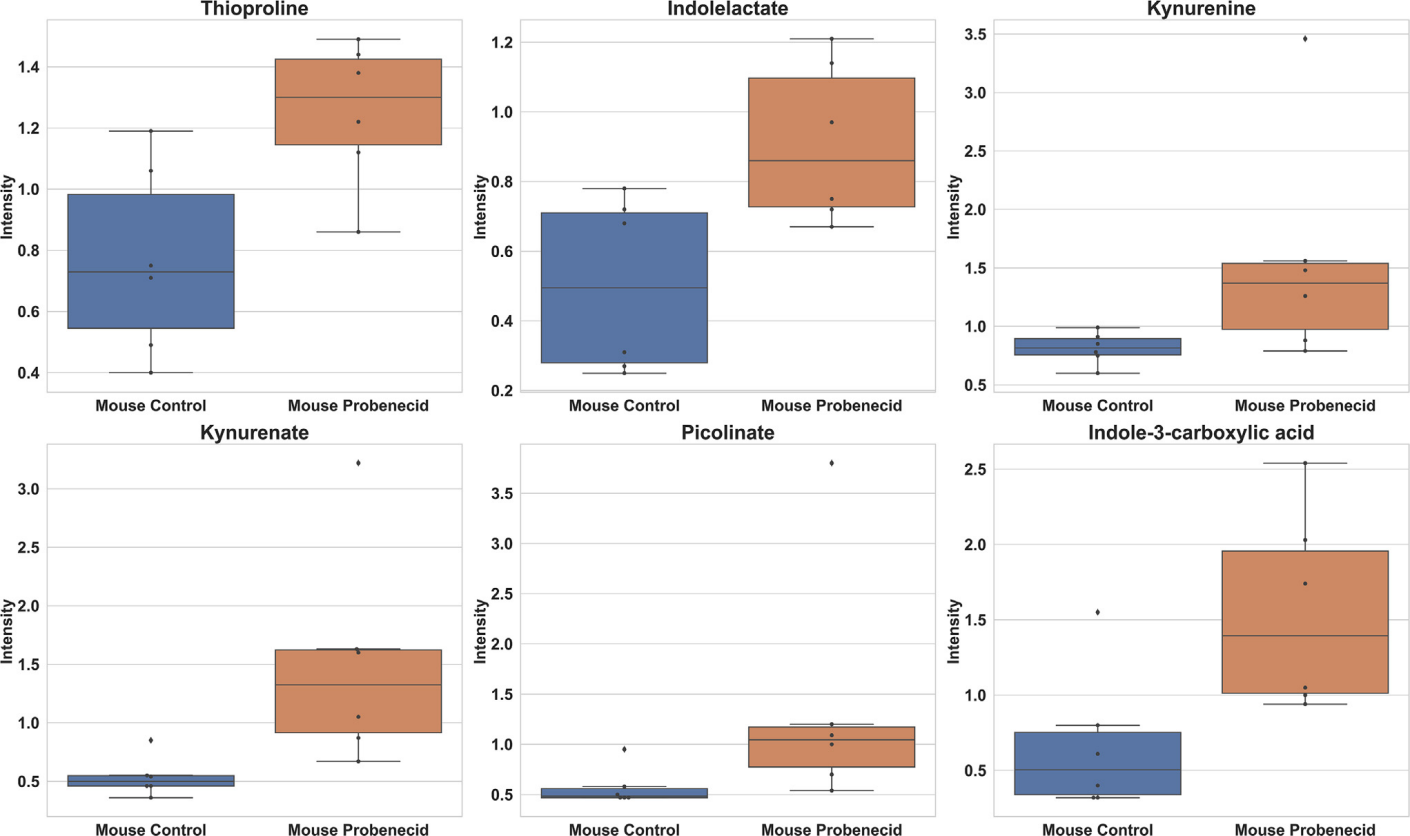
**Mice treated with probenecid had elevated circulating levels of tryptophan metabolites.** Thioproline, indolelactate, kynurenine, kynurenate, picolinate, and indole-3-carboxylic acid all demonstrated significant increases (*p* < 0.05, fold change > 1) after treatment with an OAT-inhibiting drug (n = 6).

As an intermediate between our knockout mouse models and humans, we treated wild-type mice with probenecid, a well-established OAT-inhibiting drug. This drug treatment led to the elevation of six tryptophan metabolites, including kynurenine, kynurenate, and indolelactate (Fig. 5). These metabolites were elevated in one or both knockout experiments and the probenecid-treated humans.

Thus, the overall results in probenecid-treated humans and mice, as well as the knockout mice, support the role of OAT1 and OAT3 in regulating circulating levels of tryptophan metabolites.

### Tryptophan metabolites interact with human OAT1 and OAT3 in *in vitro* transport assays

**Figure 6:**
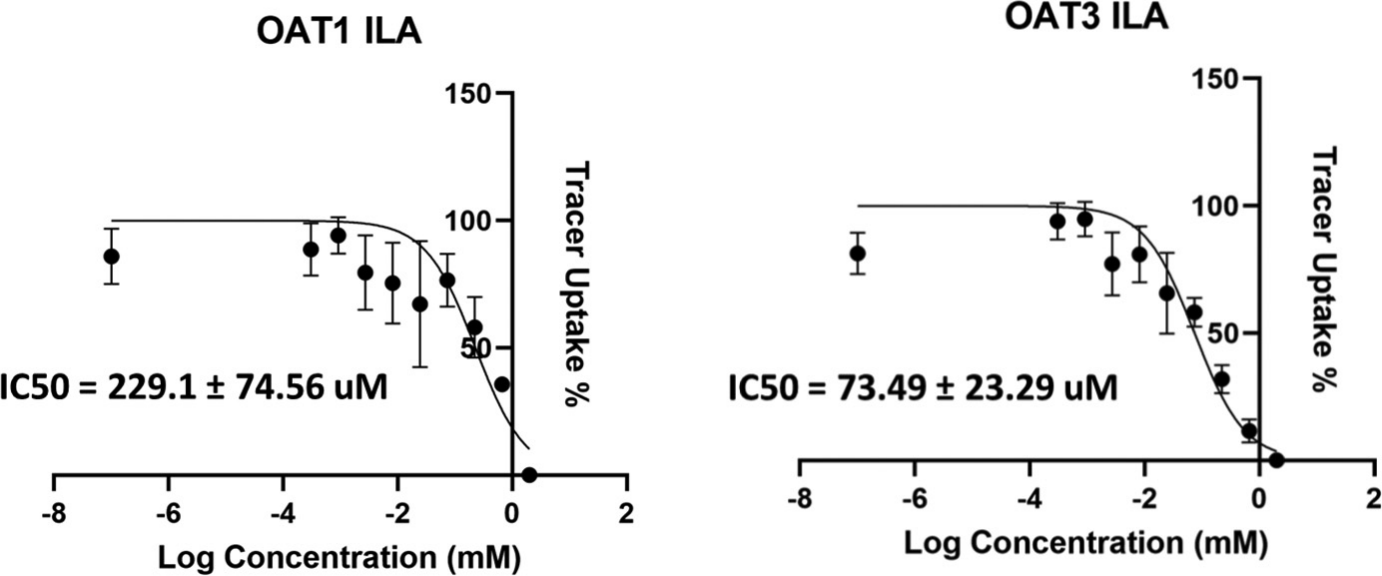
***In vitro* transport assays for tryptophan metabolites with cells overexpressing human OAT1 and OAT3.** Indolelactate (ILA) had not previously been tested for inhibition of either OAT1 or OAT3. Human OAT1 and human OAT3 transport is inhibited by ILA. Assays were repeated at least three times for each metabolite–transporter pair.

To confirm our findings, we performed *in vitro* transport assays using cells overexpressing human OAT1 and OAT3. Many of the metabolites in this Subpathway have been tested against these transporters (Table 3), but other metabolites remain unexplored. Transport assays for indolelactic acid (ILA) revealed that ILA interacts with OAT1 and OAT3 (IC50 = 229.1 ± 74.56 μM, 74.49 ± 23.29 μM respectively) (Fig. 6). Furthermore, serotonin, which was uniquely elevated in the *Oat3* KO, interacted with only OAT3 *in vitro* (IC50 = 288.2 ± 167.0 μM) (data not shown). The IC50 values were then integrated with known Km values to calculate inhibitory constants. The inhibitory constant (Ki) for ILA with OAT1 was 119.3 μM.

### Knockout kidney metabolomic task analysis indicates a pivotal role for OAT1 in proximal tubule sensing of tryptophan metabolites

We used microarray data from the kidneys of *Oat1* KO, *Oat3* KO, and their wild-type controls to identify how the transporter alterations influence the kidney metabolic functions. To this end, we used a systems biology method (metabolic task analysis (41) from the CellFie tool) that predicts how changes in gene expression impact a predefined list of 175 metabolic tasks (Supplementary Table S4), covering the general metabolic systems of a cell (energy, nucleotide, carbohydrates, amino acid, lipid, vitamin and cofactor, and glycan metabolism). As described in Methods, the computation of a “metabolic task score” takes transcriptomic data and attributes a gene activity score for each gene. A genome-scale model of metabolism is used to compile a list of reactions required to accomplish each of the 175 metabolic tasks. Thus, transcriptomic analysis can be directly used to quantitatively compare the relative activity of each metabolic function under the various conditions (*e.g.*, wild-type *versus Oat1* KO, wild-type *versus Oat3* KO).

**Figure 7:**
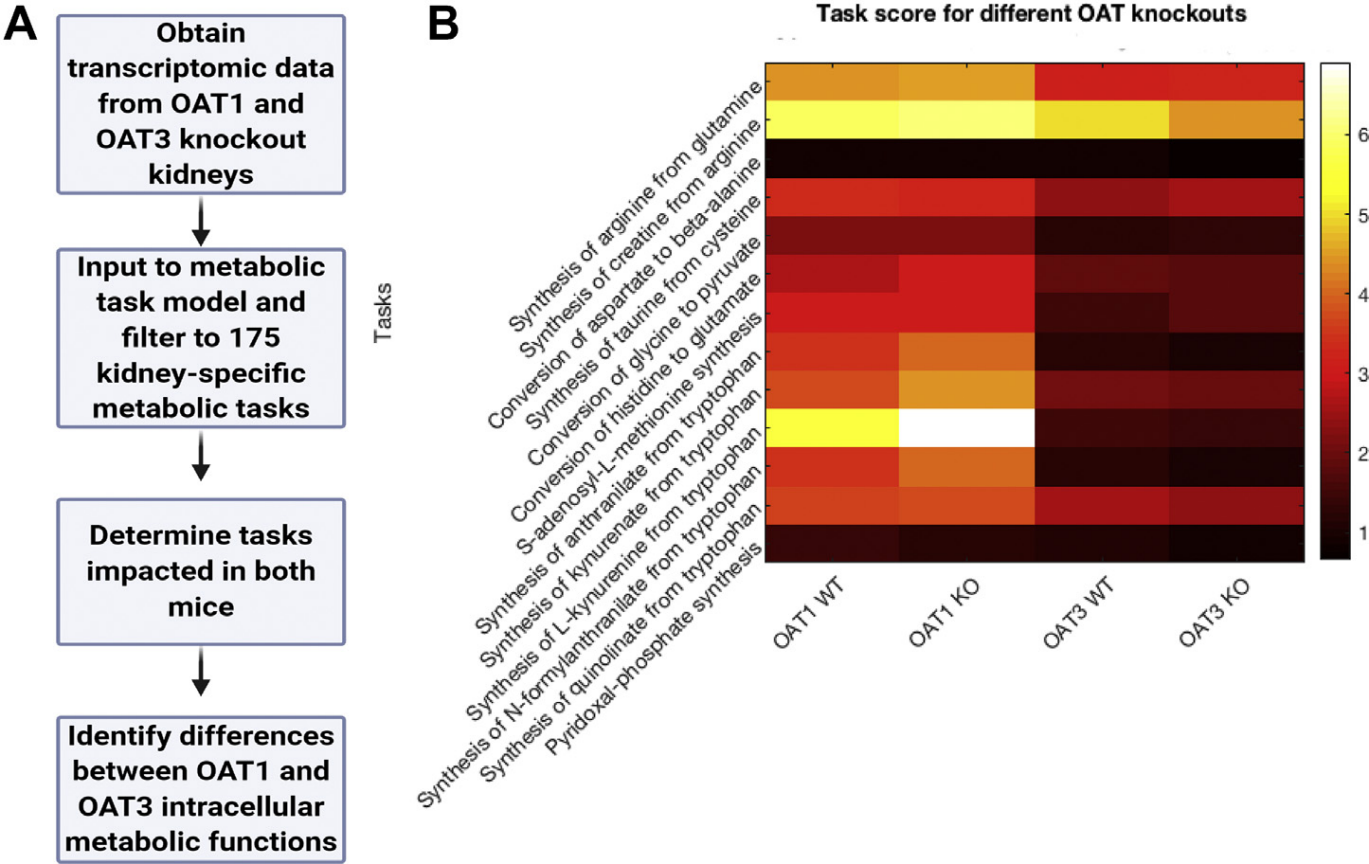
**Metabolic task analysis of WT *versus* knockout mouse kidney transcriptomics indicates OAT1 (but not OAT3) dependence of five tryptophan-related metabolic tasks.***A*, the metabolic task analysis uses transcriptomic data to predict cellular functions. *B*, the 13 tasks displayed have a coefficient of variation (standard deviation/mean) above 15% and a mean metabolic task score greater than 0.5 across both knockouts. Included in these are five tryptophan-related tasks (synthesis of anthranilate, synthesis of kynurenate, synthesis of kynurenine, synthesis of N-formylanthranilate, and synthesis of quinolinate) that have increased metabolic task scores in the *Oat1* KO (n = 3) compared with wild type (n = 3) and decreased metabolic task scores in the *Oat3* KO (n = 3) compared with wild type (n = 3). A score of 0 suggests low metabolic activity and a score of 7 suggests high metabolic activity.

While *in vitro* transport assays and other analyses have shown that OAT1 and OAT3 interact with metabolites in each of the general systems (5), we observed only few changes in the activity of most metabolic tasks between the wild-type and KO mice. However, a subset of 13 metabolic tasks exhibited a coefficient of variation over 15% between the wild-type and *Oat* KO mice (Fig. 7).

We found that tryptophan-related metabolic tasks were common among those with a large difference between *Oat* KO and wild-type mice. What was unexpected, however, was that the kidney tryptophan-related metabolic tasks were dependent on OAT1 but not OAT3. In the *Oat1* KO mice, the synthesis of kynurenine, kynurenate, anthranilate, and N-formylanthranilate from tryptophan had increased metabolic task scores, while each of those tasks had a decreased metabolic task score in the *Oat3* KO. This is consistent with the serum metabolomics analyses, which show significant elevations in the levels of kynurenine, kynurenate, anthranilate, and N-formylanthranilate in the *Oat1* KO.

## Discussion

OAT1 and OAT3 are recognized for their role in the elimination of hundreds of drugs (42). However, *in vivo* and *in vitro* studies of OAT1 and OAT3 have shown that these proteins are involved in the transport of numerous endogenous and gut microbe-derived metabolites, uremic toxins, signaling molecules, ingested nutrients, industrial toxins, and natural products (9, 24, 26, 34, 35, 43). This also appears to be the case for other multispecific drug transporters, suggesting that their contribution to endogenous metabolism is vastly underappreciated (44).

These multispecific drug transporters are highly expressed in nearly all tissues and play a particularly critical role at epithelial and endothelial barriers between blood and other body fluids/compartments (*e.g.*, blood–brain barrier, blood–retina barrier, blood–CSF barrier, nose–brain barrier, blood–urine barrier). As regulators of systemic metabolism, as well as local metabolism within their tissue of expression, the type of studies and analyses we have described here, if performed for all “drug” transporters, may radically alter our physiological views of these evolutionarily conserved proteins.

The clinical pharmaceutical importance of these transporters is immense, and regulatory agencies have recommended screening new drug entities against at least seven multispecific drug transporters (OAT1, OAT3, OATP1B1, OATP1B3, OCT2, P-gp, BCRP) to limit the possibility of drug–drug interactions (DDI)—cases where two or more drugs compete for access to the same transporter. Transporter-mediated DDIs can alter the concentration of the drugs in the blood, potentially leading to adverse clinical effects (45). Considering the number of substrates for OAT1 and OAT3, a similar phenomenon may occur between drugs and metabolites in circulation. These DMIs also have the potential to impact tissue function, as metabolites and signaling molecules may be unable to enter the cell and exert their effects on normal cellular physiology. The present study supports this possibility.

In our metabolomics analyses of the serum of *Oat1* and *Oat3* knockout mice, there were marked alterations in tryptophan-related metabolites. The increases in systemic levels of metabolites, due to the absence of these transporters at the blood interface with the kidney, raised the possibility of diminished intracellular concentration in kidney proximal tubule cells. How might these cells in turn respond? We used transcriptomics-based metabolic task analysis to address this question. We identified multiple local (kidney) tryptophan-related metabolic tasks affected by the absence of the transporters. These changes in metabolic task scores are caused by compensatory increases in the expression of the metabolic task-associated genes in the context of OAT1 deficiency. While many metabolic pathways were surveyed by both metabolomics and transcriptomics-based metabolic task analysis, the use of both approaches provided us with unique insights into how certain drug transporters participate in the communication between the tissue and the extracellular environment. Thus, whereas tryptophan metabolites were regulated by both transporters at the systemic level, tryptophan-related metabolic tasks within the kidney were primarily dependent upon OAT1.

The results indicate that the proximal tubule of the kidney, where OATs are found, is not simply a conduit for renal elimination of tryptophan metabolites; it senses tryptophan metabolites and responds to changes in their intracellular abundance. Collectively, the data supported the view that OAT1 plays a key role not only in clearing tryptophan-related metabolites from the circulation by promoting their uptake by the kidney, but also that this transporter regulates intracellular metabolism, notably tryptophan metabolism.

**Figure 8:**
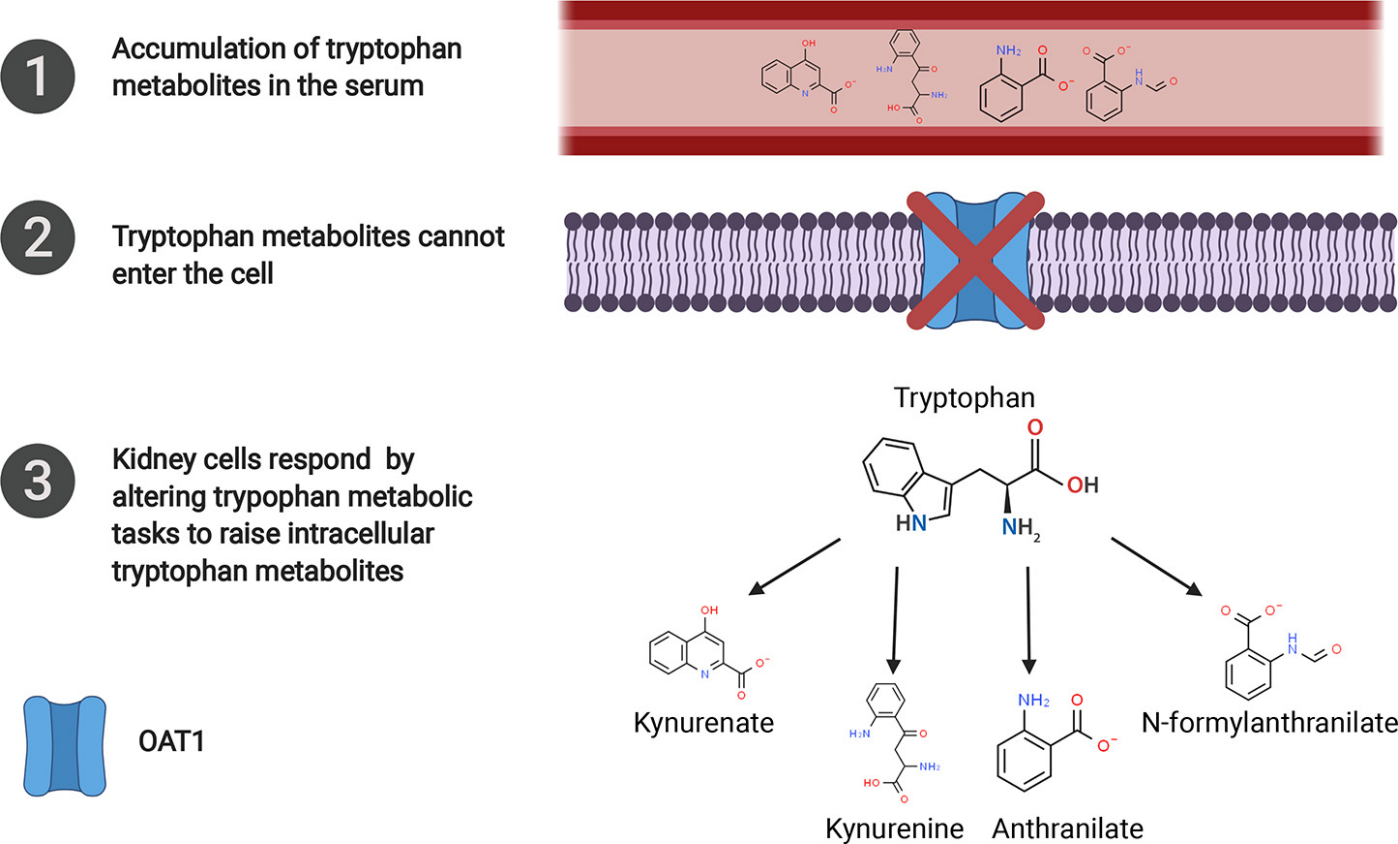
**The data predicts that kidney tissue responds to the increases in serum concentration by increasing synthesis of these metabolites intracellularly.** Anthranilate, kynurenine, kynurenate, and N-formylanthranilate were elevated in the serum of the *Oat1* KO and the transcriptomic data from the knockout kidney indicates that the tissue is producing more from tryptophan. Additionally, kynurenine, kynurenate, and N-formylanthranilate were elevated in the serum of probenecid-treated humans (anthranilate was not measured), raising the possibility that a similar process may occur within humans taking drugs that inhibit OAT1 and potentially alter intracellular tryptophan metabolic tasks as in the *Oat1* knockout mouse.

This supports the view that OAT1-mediated transport of kynurenine, kynurenate, anthranilate, and N-formylanthranilate are important for kidney function (Fig. 8). These four metabolites belong to the kynurenine subpathway of tryptophan degradation, and barring kynurenate, they are involved in the production of cellular energy through the synthesis of NAD+ (46). Thus, it is possible that the lack of OAT1 leads to impaired cellular metabolism that can be, at least in part, recovered through the production of these metabolites.

Nearly all ingested tryptophan is metabolized into three main subpathways: kynurenine, serotonin, and indole (47, 48). In addition to its role in producing NAD+, the kynurenine subpathway produces metabolites that have a variety of functions in both healthy and diseased states (46, 49). The serotonin pathway produces the neurotransmitter, serotonin, which has a role in numerous physiological processes, most notably as a regulator of CNS function (50). The indole subpathway, which is mediated by the gut microbiome, produces signaling molecules that participate in host–microbial communication (51). Systemically, OAT1 and OAT3 modulate the bioavailability of tryptophan metabolites from each of the three subpathways, though most of the metabolites come from the kynurenine and indole subpathways.

Considering the myriad of signaling roles the elevated metabolites have, there is potential for OAT1 and OAT3 to influence many aspects of physiology. For example, kynurenate activates GPR35, a drug target and a GPCR involved in inflammatory responses and cardiovascular disease (52). Many of the significantly altered tryptophan metabolites are also established or putative ligands of the aryl hydrocarbon receptor (AhR) (Table 3), a transcriptional regulator expressed in nearly all tissues that responds to xenobiotics (53).

The metabolomics studies of the two knockout mice showed that a key endogenous function of OAT1 and OAT3 is to regulate systemic levels of tryptophan metabolites. Thus, a drug targeting OAT1 and OAT3 would be expected to have a similar impact on the metabolome. As predicted, the translational potential of our knockout mice as models of DMIs was strongly supported by the results from humans treated with probenecid, an OAT-inhibiting drug that is used worldwide to treat gout. Several of the tryptophan metabolites were elevated in both the serum of humans and the knockout mice.

Thus, there is potential for the compounds elevated in human and rodent studies to be used as biomarkers for novel drug entities that may inhibit OAT1 and OAT3. While the knockout mouse models (which have normal life expectancy) and the probenecid-treated humans were healthy, some of the elevated metabolites are known uremic toxins that are increased in the serum of humans suffering from chronic renal failure and associated with negative outcomes (54, 55). The overlap of metabolites implicated in aspects of CKD and our studies suggested that the OATs may play a key role in manifestations of CKD or its progression, though it is also possible that metabolite concentrations may be increased due to loss of secretion as a result of tissue damage (56, 57). CKD can also lead to multiorgan failure, in part due to the accumulation of uremic toxins, and this may be partly due to uptake into other tissues *via* SLC and ABC drug transporters (12, 58).

The tryptophan metabolism pathway requires the coordinated function of several organs. Tryptophan is absorbed by the gut, modified by the liver or other organs, and the metabolites are ultimately transported by the kidney, in part through the function of OAT1 and OAT3. Conventionally, this is viewed simply as an elimination pathway. Our results, which indicate that cells of the kidney respond to the absence of these metabolites and signaling molecules by gearing up to produce them, suggest otherwise. Although their specific role in the kidney proximal tubule is poorly defined, it is clear that many of the intermediates produced are neurotransmitters and have an important role in CNS function (46, 49, 59, 60). In addition to interorgan communication, tryptophan metabolism is also reflective of interorganismal communication between the host and the gut microbiome. Indeed, the serum of germ-free mice has decreased levels of 3-indoxyl sulfate, indolepropionate, and serotonin (61). Furthermore, previous studies have shown that within renal OAT1-positive cells, 3-indoxyl sulfate functions in cell signaling by activating AhR and regulating their own secretion through OAT1 (62). Our findings that gut-derived metabolites were increased in the serum of both the *Oat1* KO and *Oat3* KO mice—together with our demonstration that some are ligands *in vitro*, provide additional support for the importance of these transporters in regulating communication between the host and commensal organisms. Altogether, our results align with the Remote Sensing and Signaling Theory, which proposes that drug transporters and drug metabolizing enzymes participate in interorgan and interorganismal communication through the transport and modification of small molecules to maintain homeostasis (63, 64). Thus, it is critical to understand the gamut of endogenous physiological functions of drug transporters systemically and locally.

With improved metabolomics data, we identified metabolites impacted by the absence of OAT1 and OAT3 in rodent models and metabolites impacted by the inhibition of OAT1 and OAT3 in humans treated with OAT-inhibiting drugs. Our group has previously used metabolic reconstruction networks to predict metabolic function and reported several shared and unique pathways regulated by OAT1 and OAT3 (43, 65). However, these studies were limited by early versions of reconstruction tools and very little metabolomics data. Here, using a different approach, Metabolic Task Analysis placed a much greater emphasis on the role of OAT1 in intracellular tryptophan metabolism of the kidney. Our approach can be applied to investigate the endogenous functions of other SLC and ABC family members and build separate but overlapping networks for all these drug transporters, which are found not only in mice but also in fly and worm (20, 66). Such representations will also facilitate understanding the full extent of DMIs for drugs interacting with OAT1, OAT3, or both, which likely go beyond simple competition at the level of the transporter.

## Experimental procedures

### Animals

All experimental protocols involving the use of animals were approved by the UCSD Institutional Animal Care and Use Committee (IACUC). All animals were handled in accordance with the Institutional Guidelines on the Use of Live Animals for Research. Adult WT, *Oat1* KO, and *Oat3* KO males were housed separately under a 12-h light–dark cycle and were provided ad libitum access to food and water. These animals have been described in previous publications (26, 27). Probenecid-treated mice were administered a daily intraperitoneal injection of 200 mg/kg probenecid or PBS for 3 days before sacrifice. The final injection was administered 2 h before sacrifice. The data sets used for this analysis have also been partially described (23, 24). The *Oat1* KO data set has been partly studied from a chemoinformatic perspective, but detailed pathway analysis was not performed (23). The *Oat3* KO data set was reanalyzed and only two groups were compared (24).

### Human studies with probenecid

All experimental protocols were reviewed and approved by the Institutional Review Board and abide by the Declaration of Helsinki Ethical Principles. Whole-blood samples were collected from 20 individuals (14 females, six males). The average age was 30.85 ± 10.98, and the average BMI was 24.18 ± 3.52. Participants were not taking any medications and were on vegetarian diets for the duration of the study. An oral dose of 1 gram of probenecid was administered, and after 5 h, whole blood was collected again. Each sample was kept frozen at –80 °C until metabolomic analysis.

### Metabolomics

Human serum samples were immediately stored at –80 °C and shipped on dry ice to Metabolon Inc. Mouse serum samples were collected and analyzed, as previously described (23, 24). To briefly recapitulate, for each sample, targeted metabolomic profiling was performed by Metabolon Inc. The MicroLab STAR system from Hamilton Company was used to prepare each sample, and several recovery standards were added for quality control. The serum was precipitated with methanol and stirred with Glen Mills GenoGrinder 2000 to remove proteins from the serum and release molecules bound to those proteins. The resulting solution was separated into four smaller samples. Two were analyzed by reverse phase (RP) ultraperformance liquid chromatography (UPLC) mass spectrometry (MS) with positive ion mode electrospray ionization (ESI). One sample was analyzed by RP/UPLC-MS/MS with negative ion mode ESI. One sample was analyzed by HILIC/UPLC-MS/MS. Organic solvent was removed by placing each sample on a TurboVap (Zymark).

### Statistics

For both human and mouse samples, raw values were normalized to volume, log transformed, and missing values were replaced with the lowest observed value for each compound. In the human serum samples, statistical significance was determined using ANCOVA contrasts that incorporate BMI and age. For mouse serum samples, significance was determined using Welch's two-sample *t*-test with metabolites that achieved statistical significance (*p* ≤ 0.05), as well as those approaching significance (0.05 ≤ *p* ≤ 0.10) included in subsequent analyses. Enrichment was determined using Equation 1, where *k* is the number of significantly altered metabolites in a Subpathway, *m* is the number of metabolites in a Subpathway, *n* is the number of significantly altered metabolites in the total data set, and *N* is the number of measured metabolites in the total data set.

(1) $$Enrichment\;=\;\frac{\frac{k}{m}}{\frac{n-k}{N-m}}$$

### Metabolic task analysis

We used the metabolic task analysis, as implemented in the CellFie module in GenePattern, to quantify the kidney's metabolic functions and the influence of OAT transporter alterations from gene expression data (41) (*i.e.*, microarray data from the kidneys of *Oat1* KO, *Oat3* KO, and their wild-type controls). This analysis predicts the activity of a curated collection of hundreds of tasks covering seven major metabolic activities of a cell (energy generation, nucleotide, carbohydrate, amino acid, lipid, vitamin and cofactor, and glycan metabolism) directly from transcriptomic data by using genome-scale models of human metabolism. More specifically, the computation of the relative activity of a metabolic task (*i.e.*, metabolic task score) relies first on the preprocessing of the available transcriptomic data and the attribution of a gene activity score for each gene (67). Genome-scale model of human metabolism is further used to identify the list of reactions required to accomplish each metabolic task and, doing so, to identify the list of genes that may contribute to the acquisition of a metabolic function based on GPR rules (*i.e.*, Gene Protein Reaction rules). Therefore, the metabolic task score is computed as the average activity score of all the genes contributing to a metabolic function. Doing so, transcriptomic data can be directly used to quantify the relative activity of each metabolic function in a specific condition.

### *In vitro* transport assays

Human embryonic kidney (HEK)-293 cells that stably overexpress human OAT1 and OAT3 (Solvo Biotechnology) were grown to confluence in Dulbecco's Modified Eagle's Medium (Invitrogen) supplemented with 10% fetal bovine serum and 1% penicillin/streptomycin and maintained in 5% CO_2_ at 37 °C. The OAT1-expressing cells were selected in the presence of blasticidin, and the OAT3-expressing cells were selected in the presence of puromycin. Both cell lines tested negative for *Mycoplasma* contamination. Prior to functional assays, cells were plated onto 96-well plates, incubated for 24 h, and supplemented with media. Competitive uptake experiments were carried out by incubating cells in buffer solution with a fixed concentration of 10 μM 6-carboxyflourescein and a serially diluted concentration of the proposed substrate beginning at 2 mM. Buffer was removed after a 10-min incubation at room temperature, and cells were rinsed with DPBS three times. The fluorescence was then assessed using a fluorescent plate reader. IC50 values were determined using GraphPad Prism 8.

Fluorescent intensity values were normalized so that the lowest value was set to 0% and the highest value was set to 100%. Following normalization, the data was fit to a nonlinear model, and the IC50 was determined using Equation 2.

(2) $$y\;=\;\frac{100}{1\;+\;10^{\left(x\;-\;LogIC50\right)}}$$

Ki was then calculated for each metabolite using the Cheng–Prusoff equation (Equation 3), with the Km for hOAT1 HEK293 cells derived from previous experiments (68).

(3) $$Ki\;=\;\frac{IC50}{1\;+\;\frac{\left[S\right]}{\left[Km\right]}}$$

### Data availability

All relevant metabolomics data are contained within the article and supplementary material. Transcriptomic data are available upon request from snigam@health.ucsd.edu.

## Supporting information

This article contains supporting information.

## Conflicts of interest

The authors declare that they have no conflicts of interest with the contents of this article.
